# The association of a combined healthy lifestyle with the risk of postmenopausal breast cancer subtypes in the Netherlands Cohort Study

**DOI:** 10.1007/s10654-023-01005-4

**Published:** 2023-05-11

**Authors:** Piet A. van den Brandt

**Affiliations:** 1 Department of Epidemiology, GROW- School for Oncology and Reproduction, Maastricht University Medical Centre, PO Box 616, 6200 MD Maastricht, The Netherlands; 2 Department of Epidemiology, CAPHRI- School for Public Health and Primary Care, Maastricht University Medical Centre, PO Box 616, 6200 MD Maastricht, The Netherlands

**Keywords:** Breast cancer, Healthy lifestyle, Cohort study, Prevention

## Abstract

The association between combined healthy lifestyle and postmenopausal breast cancer risk has been studied in various cohort studies, but only few evaluated the association with estrogen/progesterone (ER/PR) receptor subtypes of breast cancer, with inconsistent results. The relationship of a healthy lifestyle score (HLS) with risk of postmenopausal breast cancer (subtypes) was investigated in the Netherlands Cohort Study. In 1986, 62,573 women aged 55–69 years provided information on dietary and lifestyle habits. The HLS was derived from information on smoking, body mass index, physical activity, Mediterranean diet adherence, and alcohol intake. After 20.3 years of follow-up, multivariable case-cohort analyses were based on 2321 incident breast cancer cases, and 1665 subcohort members with complete data on lifestyles and confounders. The HLS showed a statistically significant inverse relationship with postmenopausal breast cancer risk, in a linear fashion. A one-point increment of the HLS was accompanied by a Hazard Ratio (HR) reduction of 20% for overall breast cancer. The associations between HLS and risk of ER/PR breast cancer subtypes were also significantly inverse, except for ER- breast cancer where the inverse association did not reach statistical significance. Per HLS-increment of one point, the HR reduction ranged from 14% for ER-breast cancer to 29% for ER + PR- breast cancer. These findings suggest that adhering to a combination of healthy modifiable lifestyle factors may substantially reduce the risk of overall postmenopausal breast cancer and its hormone receptor subtypes.

**Supplementary Information:**

The online version contains supplementary material available at 10.1007/s10654-023-01005-4.

## Introduction

Breast cancer is the most commonly diagnosed cancer in Western countries, and prevention is of great importance to reduce the burden of this disease. For postmenopausal breast cancer, multiple risk factors have been established [1], such as reproductive factors (e.g., age at menarche, age at menopause, parity, age at first birth), family history of breast cancer, body height, and history of benign breast disease. While these factors are generally nonmodifiable, few modifiable (lifestyle) risk factors have been identified, such as overweight, low physical activity and alcohol consumption [1]. Intake of individual dietary factors has been extensively studied in relation to breast cancer risk, but only for alcohol there is convincing evidence for an increased risk [2]. However, recent evidence suggests an inverse association between healthy dietary patterns (e.g., Mediterranean diet) and breast cancer risk [3].

General cancer prevention guidelines concerning healthy lifestyle include modifiable factors such as tobacco smoking, alcohol consumption, diet, physical activity, and relative weight (Body Mass Index, BMI). While the individual roles of these lifestyle factors in cancer risk have been extensively documented [2, 4, 5], little is known about their joint effects. Studying combinations of lifestyle factors in relation to cancer risk seems more fruitful, thereby acknowledging interactions between individual factors as well as existing collinearity between factors. Prospective studies that investigated combinations of the abovementioned modifiable lifestyle factors have reported a reduced risk of total cancer [6] when comparing subjects with more healthy lifestyles to those with less healthy lifestyles.

The association between combined healthy lifestyle and postmenopausal breast cancer risk has been studied in various cohort studies [7–22]. However, only few of these evaluated the association between combined healthy lifestyle and estrogen/progesterone (ER/PR) receptor subtypes of breast cancer, with inconsistent results [11, 14, 15, 17].

The impact of a healthy lifestyle score, combining information on smoking, BMI, physical activity, alcohol intake, and Mediterranean diet adherence, on the risk of postmenopausal breast cancer (subtypes) was investigated in the Netherlands Cohort Study (NLCS). In the NLCS, a healthy lifestyle score (HLS) consisting of these factors was strongly inversely related to risk of esophageal and gastric cancer (subtypes) [23], as well as overall mortality [24]. In the NLCS, each of the abovementioned lifestyle factors was related to breast cancer risk [25–29]. The aim of the current investigation was to evaluate the association between the healthy lifestyle score and risk of breast cancer overall, and hormone receptor subtypes.

## Methods

### Study design and cancer follow-up

The NLCS started in September 1986 and the female part included 62,573 women aged 55–69 years [30]. At baseline, participants completed a mailed, self-administered questionnaire on cancer risk factors. The NLCS study was approved by institutional review boards from Maastricht University and the Netherlands Organization for Applied Scientific Research. All cohort members consented to participation by completing the questionnaire. For efficiency, we applied the nested case-cohort method [31], requiring only data-entry of questionnaires (which could not be scanned) of cases and a random subcohort. Following this method [30], cases were enumerated from the entire NLCS-cohort of 62,573 (numerator information of incidence rates), whereas the accumulated person-years at risk in the cohort were estimated using a subcohort of 2589 women (denominator information). The case-cohort method implies that the persontime at risk is estimated through a sample of the total cohort, instead of actively following the total cohort. Data entry of questionnaires is only needed for cases and subcohort members, instead of the total cohort [30, 31]. Immediately after the NLCS-baseline measurement, the subcohort (2589 women) was randomly sampled from the cohort, and actively followed up since 1986 for vital status and migration. The follow-up of the subcohort was 100% complete at 20.3 years of follow-up.

Follow-up for cancer incidence in the entire cohort was established by annual record linkage with the Netherlands Cancer Registry and PALGA, the nationwide Dutch Pathology Registry [32]. Completeness of follow-up through record linkage with cancer registries and PALGA was estimated to be greater than 95% [33]. After 20.3 years of follow-up (September 17, 1986 until January 1, 2007), a total of 3354 incident breast cancer cases were detected among women. Cases and subcohort members were excluded if they reported a history of cancer (except skin cancer) at baseline. Furthermore, participants with incomplete or inconsistent dietary data [34], or missing values for the other considered lifestyle factors and predefined confounders were excluded from the analysis. Figure S1 (Supplementary data) shows the selection and exclusion steps that resulted in the number of postmenopausal breast cancer cases (including subtypes defined by hormone receptor status) and female subcohort members that were included in the analysis. There were 1665 subcohort members and 2321 breast cancer cases available for analysis.

### Exposure assessment

The 11-page baseline questionnaire measured dietary intake (including alcohol), detailed smoking habits, anthropometry, physical activity and other risk factors related to cancer [30]. Habitual consumption of food and beverages during the year preceding baseline was assessed using a 150-item semi-quantitative food-frequency questionnaire. The food-frequency questionnaire has been validated and tested for reproducibility [34, 35]. Nutrient intakes were calculated using the computerized Dutch food composition table [36]. Consumption of alcoholic beverages was addressed by questions on beer, red wine, white wine, sherry and other fortified wines, liqueur types containing on average 16% ethanol, and (Dutch) gin, brandy, and whiskey. Respondents who consumed alcoholic beverages less than once a month were considered non-users. Tobacco smoking was addressed through questions on smoking status (never, ex, or current smoker) and inhalation for cigarette, cigar, and pipe smokers. Additional questions were asked on the ages at first and last exposure to smoking, smoking frequency, and duration for cigarette, cigar, and pipe smokers. Information on height (in cm) and weight at baseline (in kg) was also collected using the self-administered questionnaire, from which BMI (weight/height^2^) was calculated in kg/m^2^. Non-occupational physical activity was calculated by adding the minutes spent per day on cycling or walking, shopping, walking the dog, gardening, and sports or exercise as reported previously [27].

### Mediterranean diet score

Adherence to the MD was assessed using the alternate Mediterranean Diet Score (aMED) [37, 38], which is an adapted version of the traditional Mediterranean Diet Score created by Trichopoulou et al. [39] [40]. The aMED contains 9 dietary components that are typical of the Mediterranean diet. To control for energy intake, the intake of each component was first adjusted to a daily intake of 2000 kcal [37, 38, 40]. For each of the presumed beneficial food items (vegetables (without potatoes), legumes, fruits, nuts, whole grains, fish, and the ratio of monounsaturated to saturated fatty acid intake (MUFA:SFA)), one point was given when the intake was at least the sex-specific median intake, and zero otherwise. For red and processed meat, 1 point was given (and 0 otherwise) when the intake was below the sex-specific median intake. In the full aMED, 1 additional point is normally given when alcohol intake is between 5–25 g/day, and 0 otherwise [38]. However, since alcohol is a risk factor for breast cancer, alcohol was excluded from the aMED score. The reduced 9-point sum score (aMEDr) ranged from zero to eight points (minimal to maximal conformity).

### Healthy lifestyle score

As in a previous analysis of lifestyle factors and risk of esophageal and gastric cancer subtypes [23], a combined healthy lifestyle score was constructed. In this healthy lifestyle score (HLS), scores for five modifiable lifestyle factors (BMI, smoking, physical activity, Mediterranean diet adherence, and alcohol intake) were combined, while each component factor was scored on three levels, representing full (score 1), partial (0.5) and noncompliance (0) with the public health recommendation for that component. Table 1 shows the cutoffs and scores for each of these five factors. The cutoffs for physical activity and alcohol were in line with recommendations from the Health Council of the Netherlands [41, 42]. The combined sum score ranged from zero to five points (minimal to maximal healthy lifestyle).

**Table 1:** Definition of the combined healthy lifestyle score (HLS), representing full, partial and noncompliance with public health recommendations

Lifestyle factor	Compliance with public health recommendations		
	Full (1 point)	Partial (0.5 point)	No (0 point)
BMI (kg/m^2^)	18.5- < 25 (normal weight)	25– < 30	≥ 30 or < 18.5
Smoking status	Never smoker	Former smoker	Current smoker
Physical Activity	Nonoccup PA > 60 min/day	> 30– ≤ 60 min/day	≤ 30 min/day
Diet: aMEDr	aMEDr: 6–8 points (high)	4–5 points (mod)	0–3 points (low)
Alcohol	≤ 10 g/day	> 10- < 25 g/day	≥ 25 g/day

aMEDr: alternate Mediterranean Diet Score excluding alcohol (range 0–8 points)

*Minimum–Maximum score HLS: 0–5 (intervals 0.5)*

**Table 2:** Baseline characteristics (means, or percent) by combined healthy lifestyle score (HLS) in female subcohort members with complete dietary and covariable data, Netherlands Cohort Study

Characteristic	Combined healthy lifestyle score (points), categories					
	0–1.5	2	2.5	3	3.5	4–5
*Median score (pts)*	*1.5*	*2.0*	*2.5*	*3.0*	*3.5*	*4.0*
N	63	114	230	349	383	526
Age, mean (yr)	61.6	60.8	61.6	61.6	61.3	61.2
Energy intake (kcal/day)	1665	1691	1657	1696	1688	1700
Alcohol intake (g/day)	17.8	11.3	8.4	7.1	4.4	2.9
aMEDr^a^ score (points)	2.8	3.1	3.3	3.6	4.1	5.0
Physical activity, nonoccupational (min/day)	30.2	38.2	41.7	60.7	67.8	89.4
BMI (kg/m^2^)	27.0	26.6	25.8	25.4	25.0	23.8
Height (cm)	163.6	165.1	165.1	164.9	165.3	165.9
Age at menarche (years)	13.4	13.6	13.5	13.7	13.7	13.7
Age at menopause (years)	48.8	47.4	48.2	48.7	49.4	49.1
Never smoker (%)	1.6	15.8	34.3	52.1	67.4	83.3
Ever cigarette smokers only						
Smoking frequency (cigarettes/day)	16.6	13.2	11.1	11.1	9.8	9.7
Smoking duration (years)	35.5	31.6	29.7	27.0	23.5	21.6
University or higher vocational education (%)	9.5	7.0	7.4	12.0	8.6	10.8
Family history of breast cancer (%)	4.8	10.5	10.0	8.3	8.1	9.1
History benign breast disease (%)	7.9	6.1	5.2	7.7	8.9	8.9
Nulliparous (%)	19.0	14.9	19.6	19.2	18.0	17.9
Age at first birth > 30 yr (% of parous)	11.8	18.6	23.2	24.1	23.2	23.8
Ever used oral contraceptives (%)	19.0	22.8	27.4	25.2	23.8	26.8
Ever used hormone replacement therapy (%)	14.3	16.7	15.7	14.6	11.0	12.7

^a^aMEDr: alternate Mediterranean diet score excluding alcohol

**Table 3:** Hazard Ratio of breast cancer (subtypes), according to healthy lifestyle score in multivariable-adjusted^a^ analyses, Netherlands Cohort Study

	Healthy lifestyle score (HLS, points)						P-trend	Continuous, per 1 point	PAF (95% CI)
	0–1.5 pts	2	2.5 (Ref)	3	3.5	4–5			
*Breast cancer overall*									
Person-years in subcohort	920	1916	3987	5911	6734	9464		28932	
No. of cases	128	194	365	485	523	626		2321	
Multivariable-adjusted HR	1.55	1.14	1	0.90	0.83	0.70	0.001	0.80	0.193
(95% CI)	(1.05–2.29)	(0.84–1.55)		(0.71–1.14)	(0.66–1.04)	(0.56–0.87)		(0.74–0.87)	(0.107–0.271)
*ER+ breast cancer*									
No. of cases	56	107	178	230	246	304		1121	
Multivariable-adjusted HR	1.43	1.32	1	0.89	0.81	0.69	<0.001	0.79	0.200
(95% CI)	(0.90–2.26)	(0.92–1.89)		(0.67–1.17)	(0.61–1.07)	(0.53–0.89)		(0.71–0.87)	(0.095–0.293)
*ER- breast cancer*									
No. of cases	18	13	41	51	51	74		248	
Multivariable-adjusted HR	1.79	0.65	1	0.81	0.71	0.73	0.052	0.86	0.099
(95% CI)	(0.90–3.56)	(0.32–1.29)		(0.51–1.30)	(0.44–1.14)	(0.47–1.12)		(0.72–1.03)	(− 0.114–0.272)
*PR+ breast cancer*									
No. of cases	35	64	106	141	153	204		703	
Multivariable-adjusted HR	1.59	1.31	1	0.93	0.85	0.78	0.001	0.81	0.145
(95% CI)	(0.94–2.69)	(0.86–2.00)		(0.67–1.28)	(0.62–1.18)	(0.58–1.06)		(0.72–0.90)	(0.015–0.258)
*PR- breast cancer*									
No. of cases	22	33	68	72	84	96		375	
Multivariable-adjusted HR	1.38	1.00	1	0.68	0.68	0.53	<0.001	0.75	0.253
(95% CI)	(0.74–2.59)	(0.61–1.67)		(0.46–1.01)	(0.46–1.01)	(0.37–0.77)		(0.65–0.87)	(0.095–0.384)
*ER+PR+ breast cancer*									
No. of cases	35	61	102	140	150	197		685	
Multivariable-adjusted HR	1.65	1.29	1	0.96	0.87	0.78	0.001	0.81	0.153
(95% CI)	(0.97–2.80)	(0.85–1.98)		(0.69–1.33)	(0.63–1.20)	(0.58–1.07)		(0.72–0.91)	(0.022–0.266)
*ER-PR- breast cancer*									
No. of cases	15	6	30	38	35	46		170	
Multivariable-adjusted HR	1.94	0.38	1	0.77	0.63	0.58	0.020	0.81	0.192
(95% CI)	(0.90–4.18)	(0.15–0.97)		(0.45–1.33)	(0.36–1.09)	(0.34–0.97)		(0.65–1.00)	(− 0.058–0.383)
*ER+PR- breast cancer*									
No. of cases	7	27	38	34	48	49		203	
Multivariable-adjusted HR	0.82	1.55	1	0.60	0.72	0.48	<0.001	0.71	0.312
(95% CI)	(0.33–2.06)	(0.86–2.80)		(0.35–1.01)	(0.44–1.18)	(0.30–0.79)		(0.60–0.85)	(0.110–0.469)

ER, Estrogen Receptor; PR, Progesterone Receptor; HLS, healthy lifestyle score; HR, hazard ratio; PAF, population attributable fraction

^a^Multivariable analyses adjusted for: age at baseline (55–59, 60–64, 65–69 years), cigarette smoking frequency (number of cigarettes per day; continuous, centered) and duration (number of years; continuous, centered)), body height (continuous, cm), highest level of education (primary school or lower vocational, secondary or medium vocational, and higher vocational or university), family history of breast cancer in mother or sisters (no, yes), history of benign breast disease (no, yes), age at menarche (<12, 13–14, 15–16, >17 years), parity (nulliparous, 1–2, >3 children), age at first birth (<25, >25 years), age at menopause (<45, 45–49, 50–54, >55 years), oral contraceptive use (never, ever), postmenopausal hormone replacement therapy (never, ever), energy intake (continuous, kcal/day).

### Statistical analysis

The distribution of the subcohort members by the combined healthy lifestyle score and various characteristics was examined by cross-tabulations and summary statistics. Hazard ratios (HRs) and 95% confidence intervals (95% CIs) for associations of the combined healthy lifestyle score with incidence of breast cancer subtypes were estimated using Cox proportional hazards models with follow-up duration as time variable. Person-years at risk for subcohort members were calculated from baseline until diagnosis of breast cancer, death, emigration, loss to follow-up or end of follow-up, whichever came first. Standard errors were estimated using the Huber-White sandwich estimator to account for the increased variance because of subcohort sampling [43]. It was verified that the proportional hazards assumption was not violated using scaled Schoenfeld residuals [44] and -ln(-ln) survival plots.

The associations between the HLS and risk of breast cancer subtypes were investigated on a categorical and continuous scale in survival analyses. The HLS score was categorized based on the distribution in the subcohort into 6 categories: 0–1.5, 2, 2.5, 3, 3.5, and 4–5 points [23]. Participants with a HLS of 2.5 points formed the reference group in categorical analyses, because categories 0–1.5 and 2 were too small. Tests for trends were assessed by assigning median values of the lifestyle score in the subcohort to the exposure categories and fitting these as continuous terms in the regression models. In the continuous analyses, HRs were estimated per increment of 1 point.

In multivariable-adjusted survival analyses, the associations were adjusted for the following predefined (literature-based) confounders, which were included in the final multivariable-adjusted model independent of their effect on the estimated HRs: age at baseline (55–59, 60–64, 65–69 years), smoking frequency (number of cigarettes per day; continuous, centered), and duration (number of years; continuous, centered), body height (continuous, cm), highest level of education (primary school or lower vocational (low), secondary school or medium vocational (medium), and higher vocational or university (high)), total energy intake (kcal/day; continuous), family history of breast cancer in mother or sisters (no, yes), history of benign breast disease (no, yes), age at menarche (≤ 12, 13–14, 15–16, ≥ 17 years), parity (nulliparous, 1–2, ≥ 3 children), age at first birth (< 25, ≥ 25 years), age at menopause (< 45, 45–49, 50–54, ≥ 55 years), oral contraceptive use (never, ever), postmenopausal hormone replacement therapy (never, ever).

For the analyses regarding the healthy lifestyle score, the association between each of the component lifestyle factors and cancer risk was also evaluated in Cox regression analyses, while controlling for age, education, energy intake, family history of breast cancer, the abovementioned reproductive/ hormonal variables, and additionally for the other lifestyle components.

Besides overall postmenopausal breast cancer, we conducted these analyses for subtypes defined by hormone receptor status: ER + , ER-, PR + , PR-, ER + PR + , ER-PR-, and ER + PR-. The number of ER-PR + cases was too small for separate analyses. Differences in associations with the healthy lifestyle score between breast cancer subtypes were tested using a heterogeneity test [45], in which the standard error for the observed difference in rate ratios was estimated using a bootstrapping method developed for the case-cohort design [46].

To further investigate the dose–response relations between the healthy lifestyle score and risk of breast cancer subtypes, restricted cubic splines with three knots were used to graphically present the dose–response curves without making a priori assumptions about their shapes. Wald tests were performed to evaluate the linearity of these relationships.

In addition to the main analyses of the healthy lifestyle score and risk of cancer subtypes, analyses were also stratified by age, level of education, family history of breast cancer, history of benign breast disease, body height, oral contraceptive use and hormone replacement therapy. Interactions with these factors were tested using Wald tests and cross-product terms. Because of low cancer case numbers, these analyses were conducted using continuous lifestyle scores. In sensitivity analyses, analyses were repeated after excluding cancers (and person-years) occurring in the first two years of follow-up. In addition, a sensitivity analysis was conducted in which alcohol intake was categorized as 0, > 0–< 25, 25 + g/day (instead of 0–10, > 10–< 25, 25 +), to use nondrinkers as separate category.

Population attributable fractions (PAFs) were calculated [47] to estimate the potentially avoidable proportion of cancer if all participants would shift towards the healthiest lifestyle category. The STATA-command “punafcc” was used to calculate the population attributable fractions and 95% Cis [48]. Analyses were performed using Stata version 14; presented P-values are two-sided, with p < 0.05 considered as statistically significant.

## Results

The mean (SD) score of the combined healthy lifestyle score (HLS) among female subcohort members was 3.3 (0.8). Table 2 summarizes several baseline characteristics by healthy lifestyle score in subcohort members. Age and energy intake were not related to the healthy lifestyle score. While the associations between the healthy lifestyle score and alcohol intake, aMEDr score, BMI, and smoking were as expected considering the score composition, women with a high HLS were somewhat more often highly educated. Women in the lowest category of the healthy lifestyle score were shorter, and less often reported a family history of breast cancer, ever use of oral contraceptives, and an age at first birth ≥ 30 years. Benign breast disease, age at menarche or menopause, and parity showed no clear trends with the HLS.

**Fig. 1:**
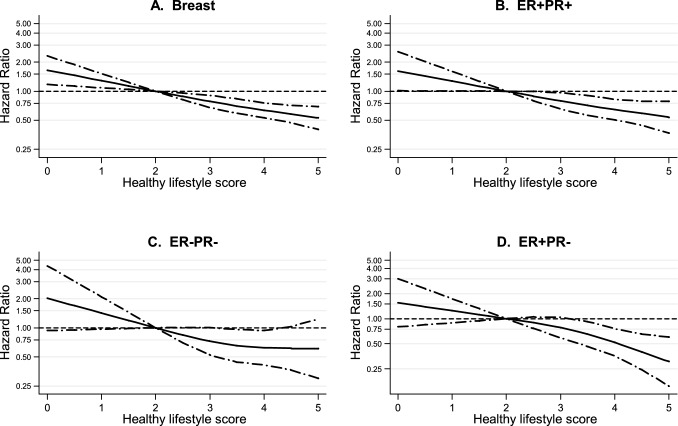
Spline regression curves for the association between healthy lifestyle score (HLS) and risk of **A** Overall breast cancer, **B** ER + PR + breast cancer, **C** ER-PR- breast cancer, and **D** ER + PR- breast cancer, Netherlands Cohort Study (NLCS). Solid lines represents point estimates and dashed lines represent 95% confidence intervals. Multivariable Hazard ratios were calculated by restricted cubic spline regression (using 3 knots) adjusting for: age at baseline (55–59, 60–64, 65–69 years), cigarette smoking frequency (number of cigarettes per day; continuous, centered) and duration (number of years; continuous, centered)), body height (continuous, cm), highest level of education (primary school or lower vocational, secondary or medium vocational, and higher vocational or university), family history of breast cancer in mother or sisters (no, yes), history of benign breast disease (no, yes), age at menarche (< 12, 13–14, 15–16, > 17 years), parity (nulliparous, 1–2, > 3 children), age at first birth (< 25, > 25 years), age at menopause (< 45, 45–49, 50–54, > 55 years), oral contraceptive use (never, ever), postmenopausal hormone replacement therapy (never, ever), energy intake (continuous, kcal/day). P-values for non-linearity tests were 0.700 for overall breast cancer, 0.820 for ER + PR + , 0.426 for ER-PR-, and 0.386 for ER + PR- breast cancer. ER, Estrogen Receptor; PR, Progesterone Receptor; HLS, healthy lifestyle score

Table 3 shows results of the multivariable-adjusted analyses of the associations of the healthy lifestyle score with risk of overall breast cancer, and its subtypes. The HLS was statistically significantly inversely associated with risk of overall breast cancer (P-trend < 0.001). Compared to women with a HLS of 2.5, the hazard ratio (HR) for women in the healthiest lifestyle category (HLS 4–5) was 0.70 (95%CI, 0.56–0.87). The HLS was also significantly inversely associated with risk of ER + , PR + , PR–, ER + PR + , ER-PR- and ER + PR- breast cancer (all with P-trend < 0.02, Table 3), whereas the association with ER- breast cancer was not significant (P-trend = 0.052). Cubic spline analyses showed no statistically significant tests for nonlinearity between HLS and risk of breast cancer or its subtypes (Fig. 1 and Supplementary Figure S2). When HLS was modelled as continuous variable in a linear fashion, this again showed statistically significant inverse associations for all outcomes, except for ER- breast cancer. Per HLS increment of 1 point, the HR for overall breast cancer was 0.80 (95%CI, 0.74, 0.87), while the HRs for the subtypes ranged from 0.71 (95%CI, 0.60, 0.85) for ER + PR- to 0.86 (95%CI, 0.72, 1.03) for ER- breast cancer (Table 3). Heterogeneity tests across subtypes using bootstrapping were not significant (data not shown). The number of ER-PR + cases was too small for separate analyses. The cumulative incidence [49] of breast cancer (calculated in accordance with the case-cohort design) is plotted against follow-up time in Supplementary Figures S3-S5.

When assuming a causal relationship between the HLS and breast cancer, estimation of the population attributable fractions (PAFs) suggested that 19.3% (95%CI, 10.7%, 27.1%) of overall breast cancer could be avoided if all participants would shift towards the healthiest HLS category (Table 3). Estimated PAFs ranged from 9.9% (95%CI, -11.4%, 27.2%) for ER- breast cancer to 31.2% (95%CI, 11.0%, 46.9%) for ER + PR- breast cancer; the PAFs are presented in Table 3.

**Fig. 2:**
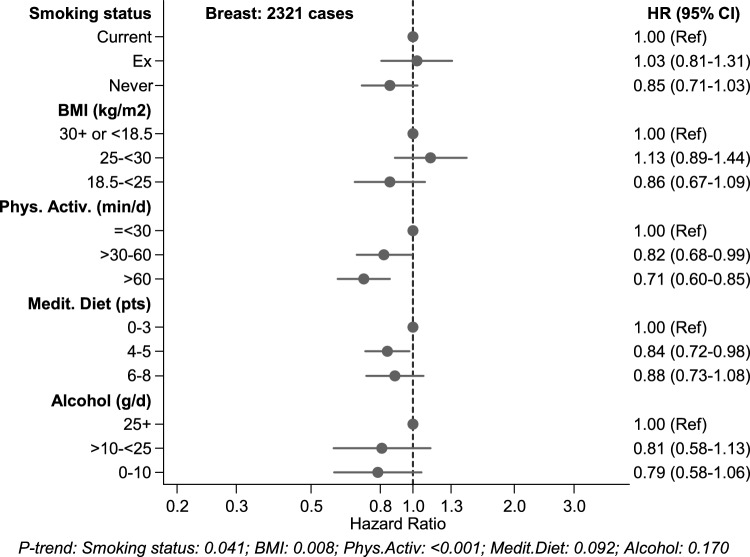
Hazard ratios and 95% CIs (error bars) for the association between overall breast cancer risk and each of the component lifestyle factors of the healthy lifestyle score (with mutual adjustment for the other component lifestyle factors), Netherlands Cohort Study (NLCS). Multivariable HRs were adjusted for: age at baseline (55–59, 60–64, 65–69 years), cigarette smoking frequency (number of cigarettes per day; continuous, centered) and duration (number of years; continuous, centered)), body height (continuous, cm), highest level of education (primary school or lower vocational, secondary or medium vocational, and higher vocational or university), family history of breast cancer in mother or sisters (no, yes), history of benign breast disease (no, yes), age at menarche (< 12, 13–14, 15–16, > 17 years), parity (nulliparous, 1–2, > 3 children), age at first birth (< 25, > 25 years), age at menopause (< 45, 45–49, 50–54, > 55 years), oral contraceptive use (never, ever), postmenopausal hormone replacement therapy (never, ever), energy intake (continuous, kcal/day), other component lifestyle factors of the HLS. ER, Estrogen Receptor; PR, Progesterone Receptor; HLS, healthy lifestyle score

**Fig. 3:**
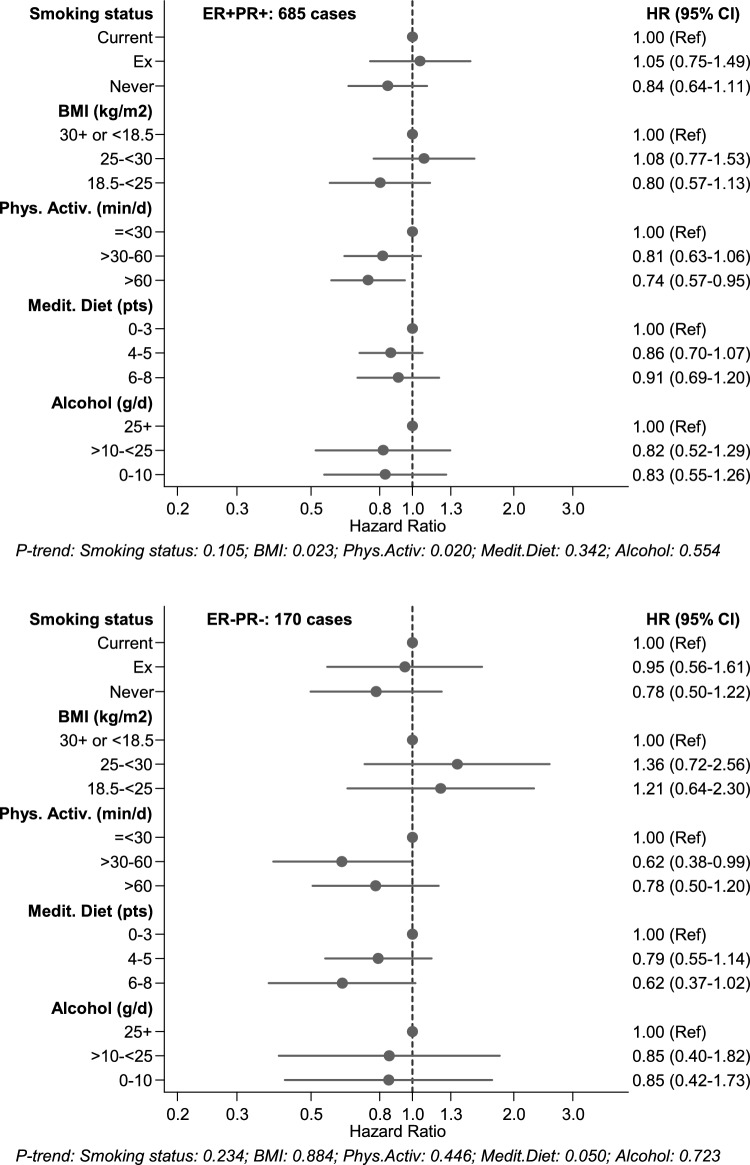
Hazard ratios and 95% CIs (error bars) for the association between risk of ER + PR + and ER-PR- breast cancer, respectively, with each of the component lifestyle factors of the HLS (with mutual adjustment for the other component lifestyle factors), Netherlands Cohort Study (NLCS). Multivariable HRs were adjusted for: age at baseline (55–59, 60–64, 65–69 years), cigarette smoking frequency (number of cigarettes per day; continuous, centered) and duration (number of years; continuous, centered)), body height (continuous, cm), highest level of education (primary school or lower vocational, secondary or medium vocational, and higher vocational or university), family history of breast cancer in mother or sisters (no, yes), history of benign breast disease (no, yes), age at menarche (< 12, 13–14, 15–16, > 17 years), parity (nulliparous, 1–2, > 3 children), age at first birth (< 25, > 25 years), age at menopause (< 45, 45–49, 50–54, > 55 years), oral contraceptive use (never, ever), postmenopausal hormone replacement therapy (never, ever), energy intake (continuous, kcal/day), other component lifestyle factors of the HLS. ER, Estrogen Receptor; PR, Progesterone Receptor; HLS, healthy lifestyle score

The multivariable-adjusted associations between each of the component lifestyle factors and breast cancer risk (with mutual adjustment for the other component lifestyle factors) are shown in Fig. 2 for overall breast cancer, and in Fig. 3 for ER + PR + and ER-PR- subtypes, and in Supplementary Figures S6-S8 for other subtypes. These analyses show that smoking was significantly associated with risk of overall breast cancer, and BMI with risk of overall and hormone receptor positive breast cancer risk. Physical activity was significantly inversely associated with risk of overall and hormone receptor positive breast cancer; MD adherence (aMEDr) was significantly inversely associated with hormone receptor negative breast cancer, while alcohol showed no statistically significant associations.

In sensitivity analyses, excluding of the first two years of follow-up did not materially change the results; the same applied to analyses with alcohol categorized as 0, > 0– < 25, 25 + g/day (data not shown).

**Fig. 4:**
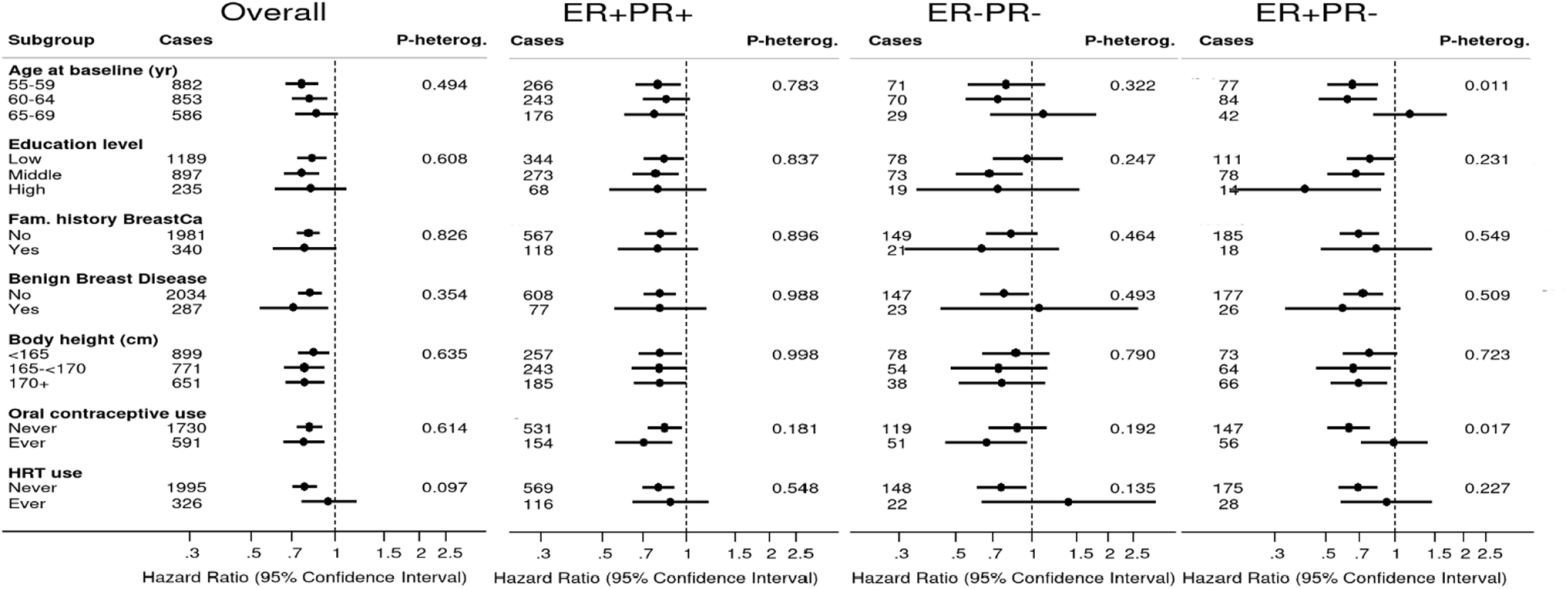
Hazard ratios and 95% CIs (error bars) of overall breast cancer (and subtypes) associated with a 1-point increment in healthy lifestyle score (HLS), in subgroups. Multivariable analyses were adjusted for: age at baseline (55–59, 60–64, 65–69 years), cigarette smoking frequency (number of cigarettes per day; continuous, centered) and duration (number of years; continuous, centered)), body height (continuous, cm), highest level of education (primary school or lower vocational, secondary or medium vocational, and higher vocational or university), family history of breast cancer in mother or sisters (no, yes), history of benign breast disease (no, yes), age at menarche (< 12, 13–14, 15–16, > 17 years), parity (nulliparous, 1–2, > 3 children), age at first birth (< 25, > 25 years), age at menopause (< 45, 45–49, 50–54, > 55 years), oral contraceptive use (never, ever), postmenopausal hormone replacement therapy (never, ever), energy intake (continuous, kcal/day). ER, Estrogen Receptor; PR, Progesterone Receptor; HRT, hormone replacement therapy

Figure 4 shows associations between a 1-point increment in HLS and overall breast cancer risk, in subgroups of potential effect modifiers: age at baseline, level of education, family history of breast cancer, history of benign breast disease, body height, oral contraceptive use and hormone replacement therapy. Inverse associations with the HLS were seen in all subgroups, and there was no significant interaction. The corresponding subgroup analyses for the subtypes ER + PR + , ER–PR–, and ER + PR– breast cancer are also presented in Fig. 4. These showed the same pattern of inverse associations for ER + PR + subtypes as for overall breast cancer; for the other subtypes the associations were often inverse but there was more variability, with statistically significant heterogeneity for age and oral contraceptive use in the ER + PR– subtype.

## Discussion

A healthy lifestyle score (HLS) which combined nonsmoking, having a normal BMI, being physically active, adhering to a Mediterranean Diet, with no or low alcohol intake, showed a strong and statistically significant inverse relationship with postmenopausal breast cancer risk in this large prospective study among Dutch women aged 55–69 years at baseline. A one-point increment of the HLS was accompanied by a HR reduction of 20% for overall breast cancer, and there was no statistical evidence of nonlinear dose–response relationships. The associations between HLS and risk of estrogen / progesterone receptor breast cancer subtypes were also significantly inverse, except for ER- breast cancer where the inverse association did not reach statistical significance. Per HLS-increment of one point, the HR reduction ranged from 14% for ER- breast cancer to 29% for ER + PR- breast cancer. These results suggest that adhering to a combination of healthy modifiable lifestyle factors may substantially reduce the risk of overall breast cancer and its hormone receptor subtypes.

The inverse association between the HLS and overall postmenopausal breast cancer risk in the NLCS is consistent with findings most other cohort studies [7–15, 17–22], but not all [16, 50]. These studies used various combinations of the factors BMI, physical activity, alcohol, one or more dietary factors, with or without smoking, in the form of the WCRF/AICR score, ACS cancer prevention guidelines score, Healthy Lifestyle Index, or other scores, but the inverse associations between healthy lifestyle and breast cancer did not seem to be limited to a particular combination score [6]. The associations between combined healthy lifestyle and hormone receptor breast cancer subtypes are less consistent in the few cohort studies that have investigated this [11, 14, 15, 17]. Although three studies [11, 14, 17] found statistically significant inverse associations with the ER + PR + subtype, the Black Women’s Health Study did not [15]. Statistically significant inverse associations with the ER-PR- subtype were found in two studies [11, 15], while associations with ER-PR- were nonsignificantly inverse in the Women’s Health Initiative Study [17], and not apparent in the Swedish Mammography Cohort [14]. The NLCS showed significantly inverse associations with both ER + PR + and ER–PR– subtypes. While the sparse available literature suggests that the inverse association seems not limited to particular hormone receptor subtypes, more studies on subtypes are needed, in particular on the possible absence of an association with ER + PR + breast cancer in black women.

A recent meta-analysis reported a summary HR (95%CI) for the healthiest versus least healthy combined lifestyle of 0.77 (0.72, 0.82) for overall breast cancer, with substantial heterogeneity in the published risk estimates [6]. In the current analysis an intermediate reference category was used, but when the healthiest combined lifestyle would be compared with the least healthy lifestyle in the NLCS, the calculated HR would become 0.45, which is on the lower side of the study-specific HRs reported in the meta-analysis [6], i.e. indicating a rather strong association. However, the reported strengths of the associations from available studies are difficult to compare, because different scoring systems and lifestyle factors are used.

In the NLCS effect-modification analysis for overall breast cancer, inverse associations with the HLS were seen in all subgroups of potential modifiers (age at baseline, level of education, family history of breast cancer, history of benign breast disease, body height, oral contraceptive use and hormone replacement therapy), and there was no significant interaction. This also applied to most hormone receptor breast cancer subtypes. Few other cohort studies have evaluated possible effect-modification, and only for overall breast cancer. As in the NLCS, Nomura et al. [13] observed no effect-modification by height, but they found an inverse association between combined healthy lifestyle and postmenopausal breast cancer only in women without a family history of breast cancer, and not among those with a positive family history of breast cancer. However, in the WHI cohort study [17], inverse associations were observed in women with and without a positive family history of breast cancer, as was also seen in the NLCS. In the same WHI cohort, no significant effect-modification by hormone therapy use or ethnicity was seen [17]. Differences between white and black women regarding associations of healthy lifestyle with overall breast cancer were also not observed in other cohort studies [15, 51].

Our PAF estimate (19.3%) for overall breast cancer associated with adhering to a healthy lifestyle is (somewhat) higher than in other cohort studies [10, 52]. In the French E3N cohort study, the PAF was 6.3% for postmenopausal breast cancer, using an index combining smoking, BMI, physical activity, alcohol, and fruits and vegetables [10], while it was 15% for “breast and reproductive” cancers in the European EPIC study using the Healthy Lifestyle Index [52]. No studies seem to have reported PAFs associated with healthy lifestyle for breast cancer subtypes until now; in the NLCS these varied from 9.9% for ER– to 31.2% for ER + PR- breast cancer. Further investigation of differences according to subtype in other large-scale studies is needed. In another type of analysis -in which the PAF is calculated by combining the HRs and observed prevalence of the risk factors- from the US Nurses’ Health Study, Tamimi et al. [53] found that the PAF for modifiable breast cancer risk factors (weight gain, alcohol, physical activity, breastfeeding, menopausal hormone therapy) was 34.6% overall, and higher for ER + than for ER- breast cancer. In the NLCS, the PAF for ER + subtype was also higher than for ER- breast cancer, but the opposite was seen for PR subtypes. The large variation seen in PAFs associated with combined HLS from different studies can be due to choice of (the number of) considered risk factors in the combination scores, the scoring system and how extreme the chosen categories for least and most healthy lifestyle were defined, and the distribution of the risk factors in the populations studied. This makes it difficult to directly compare PAFs from different studies.

In the NLCS, the component risk factors showed varying associations with risk of breast cancer (subtypes), after mutual adjustment for the remaining components, with notable differences between disease subtypes. Smoking was significantly positively associated with risk of overall breast cancer. BMI showed positive associations with risk of overall and hormone receptor positive breast cancer risk, while physical activity was significantly inversely associated with these disease types. Adherence to Mediterranean diet (aMEDr) was significantly inversely associated with hormone receptor negative breast cancer, while alcohol showed no statistically significant associations. Overall, these findings are in line with the literature [3, 54-57]. While these factors (smoking, BMI, physical activity, alcohol intake, Mediterranean diet adherence) generally showed weak or modest associations with breast cancer risk—depending on ER/PR subtype-, the additive protective effect of the factors combined was stronger than those of the individual factors in the NLCS, as was also observed in other studies (e.g., [21]).

The prospective design and high completeness of follow-up of the NLCS make information bias and selection bias unlikely. The large population-based cohort with long follow-up enabled cancer subtype-specific analyses. The NLCS also has several limitations. A potential weakness is the moderate proportion of breast cancer cases for whom ER/PR status was known. Breast cancer cases with known and unknown receptor status did not differ importantly according to baseline and tumor characteristics, making selection bias of the cases unlikely (data not shown). Although we adjusted for a large number of potential confounders, residual confounding by unmeasured factors may still exist. The validation study of the food frequency questionnaire has shown that it performs relatively well [34], but measurement error may still have attenuated associations. Changes is lifestyle factors during follow-up may have led to non-differential misclassification and attenuated associations. However, there was no possibility to update lifestyle data during follow-up.

In conclusion, this cohort study showed that adherence to a combined modifiable healthy lifestyle is significantly inversely related to the risk of overall postmenopausal breast cancer and its hormone receptor subtypes. Per HLS-increment of one point, the HR reduction ranged from 14% for ER- breast cancer to 29% for ER + PR- breast cancer. This suggests that important gains in postmenopausal breast cancer prevention can be made by adhering to a healthy lifestyle.

## Supplementary Information

Below is the link to the electronic supplementary material.

Supplementary file1 (PDF 530 kb)
