# Supplementary material for: The association of a combined healthy lifestyle with the risk of postmenopausal breast cancer subtypes in the Netherlands Cohort Study
Source: Eur J Epidemiol. 2023 May 11;38(6):629–41. doi: 10.1007/s10654-023-01005-4 (PMC10232625; doi:10.1007/s10654-023-01005-4)
Supplement: Supplementary file 1 — Supplementary file1 (PDF 530 kb) [file 10654_2023_1005_MOESM1_ESM.pdf]

SUPPLEMENTARY MATERIALS to:

European Journal of Epidemiology

Article:

P.A. van den Brandt "The association of a combined healthy lifestyle with the risk of postmenopausal breast cancer subtypes in the Netherlands Cohort Study"

Supplementary Figure S1. Flow diagram of the number of subcohort members and cancer cases on which analyses are based, Netherlands Cohort Study (NLCS).

Supplementary Figure S2. Spline regression curves for the association between healthy lifestyle score (HLS) and risk of A) ER+ breast cancer, B) ER- breast cancer, C) PR+ breast cancer, and D) PR- breast cancer, Netherlands Cohort Study.

Supplementary Figure S3. Cumulative incidence (proportion) of overall breast cancer according to healthy lifestyle score, Netherlands Cohort Study.

Supplementary Figure S4. Cumulative incidence of overall, ER+PR+, ER-PR- and ER+PR- breast cancer according to healthy lifestyle score (dichotomized), Netherlands Cohort Study.

Supplementary Figure S5. Cumulative incidence of ER+, ER-, PR+ and PR- breast cancer according to healthy lifestyle score (dichotomized), Netherlands Cohort Study.

Supplementary Figure S6. Multivariable Hazard ratios and 95% CIs (error bars) for the association between risk of ER+PR- breast cancer and each of the component lifestyle factors of the HLS (with mutual adjustment for the other component lifestyle factors), Netherlands Cohort Study.

Supplementary Figure S7. Multivariable Hazard ratios and 95% CIs (error bars) for the association between risk of ER+ and ER- breast cancer, respectively, with each of the component lifestyle factors of the HLS (with mutual adjustment for the other component lifestyle factors), Netherlands Cohort Study.

Supplementary Figure S8. Multivariable Hazard ratios and 95% CIs (error bars) for the association between risk of PR+ and PR- breast cancer, respectively, with each of the component lifestyle factors of the HLS (with mutual adjustment for the other component lifestyle factors), Netherlands Cohort Study.

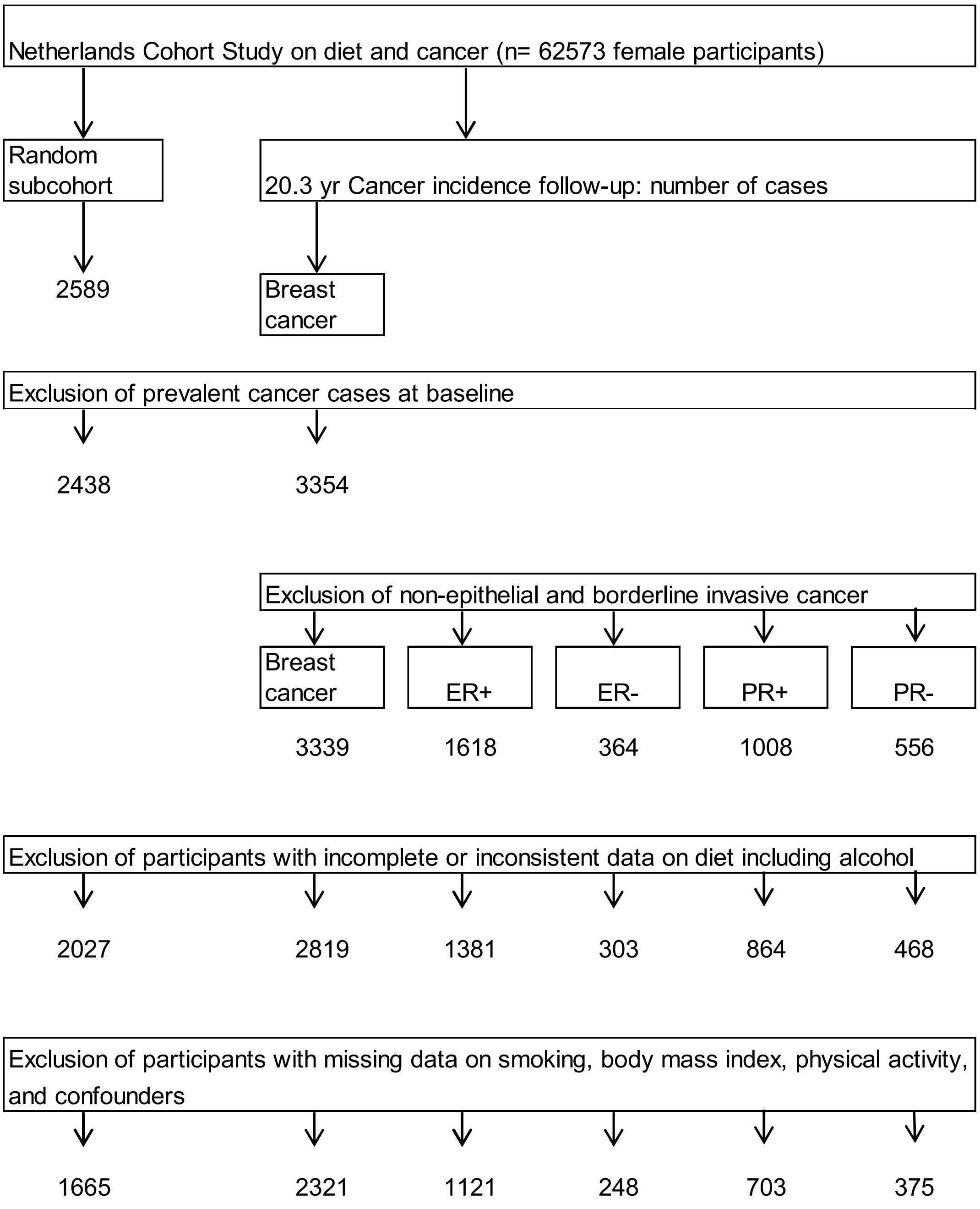

Abbreviations: ER, Estrogen Receptor; PR, Progesterone Receptor

Supplementary Figure S1. Flow diagram of the number of subcohort members and cancer cases on which analyses are based, Netherlands Cohort Study (NLCS). The final numbers of combined ERPR cases available for multivariable-adjusted analyses were: n=685 for ER+PR+, 170 for ER-PR-, 203 for ER+PR-, and 18 for ER-PR+ subtype.

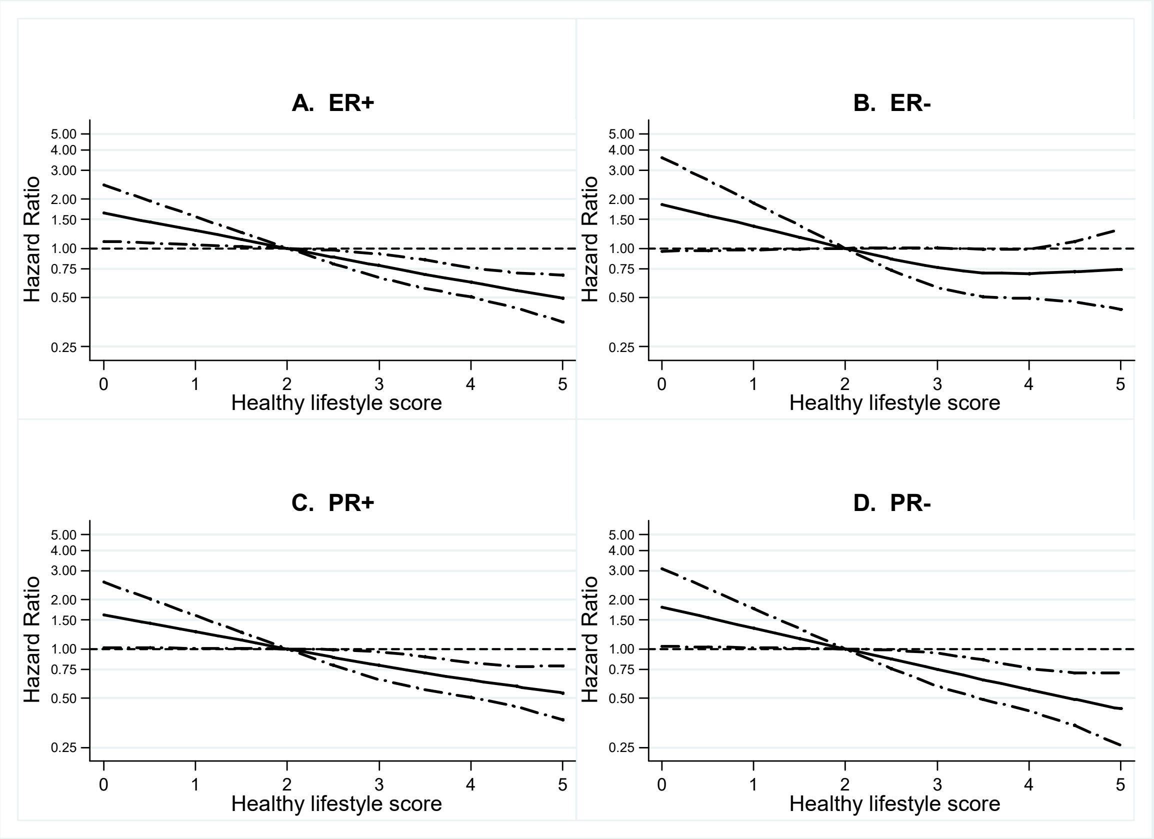

Supplementary Fig S2. Spline regression curves for the association between healthy lifestyle score (HLS) and risk of A) ER+ breast cancer, B) ER- breast cancer, C) PR+ breast cancer, and D) PR- breast cancer, Netherlands Cohort Study (NLCS). Solid lines represents point estimates and dashed lines represent 95% confidence intervals.

Multivariable Hazard ratios were calculated by restricted cubic spline regression (using 3 knots) adjusting for: age at baseline (55-59, 60-64, 65-69 years), cigarette smoking frequency (number of cigarettes per day; continuous, centered) and duration (number of years; continuous, centered)), body height (continuous, cm), highest level of education (primary school or lower vocational, secondary or medium vocational, and higher vocational or university), family history of breast cancer in mother or sisters (no, yes), history of benign breast disease (no, yes), age at menarche (<12, 13-14, 15-16, >17 years), parity (nulliparous, 1-2, >3 children), age at first birth (<25, >25 years), age at menopause (<45, 45-49, 50-54, >55 years), oral contraceptive use (never, ever), postmenopausal hormone replacement therapy (never, ever), energy intake (continuous, kcal/day).

P-values for non-linearity tests were 0.919 for ER+ breast cancer, 0.273 for ER-, 0.798 for PR+, and 0.948 for PR- breast cancer. *Abbreviations:* ER, Estrogen Receptor; PR, Progesterone Receptor; HLS, healthy lifestyle score.

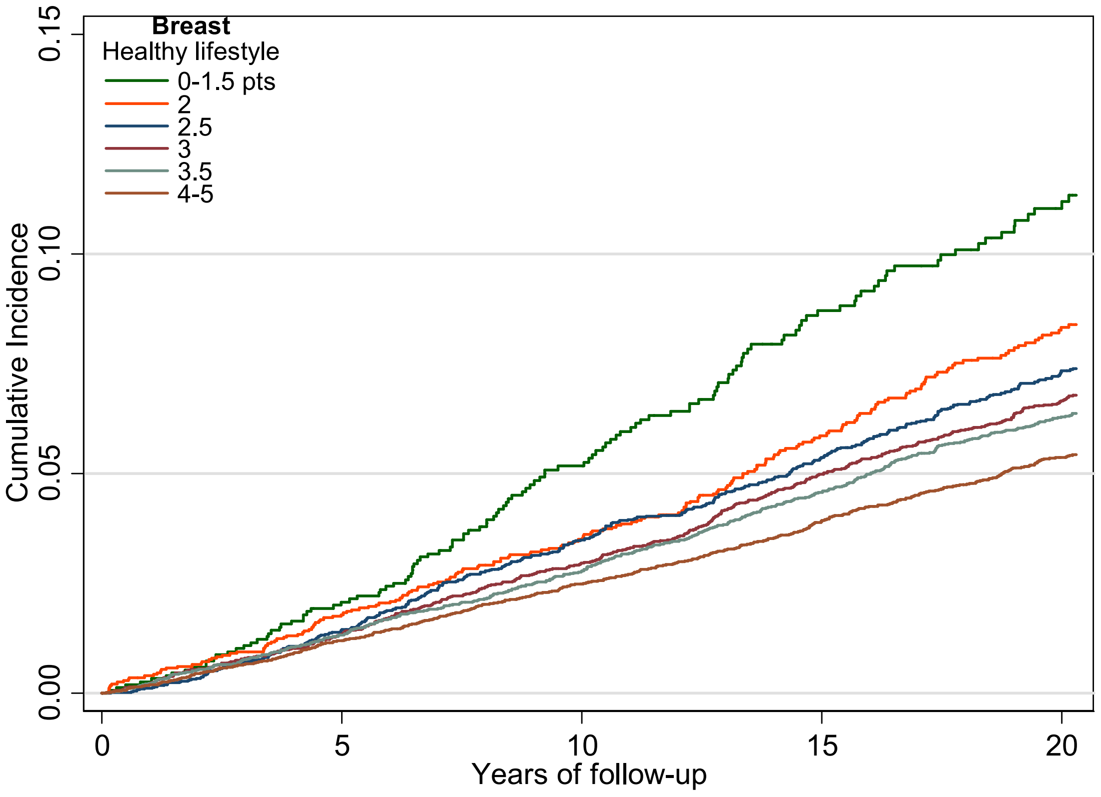

Supplementary Figure S3. Cumulative incidence (proportion) of overall breast cancer according to healthy lifestyle score, Netherlands Cohort Study.

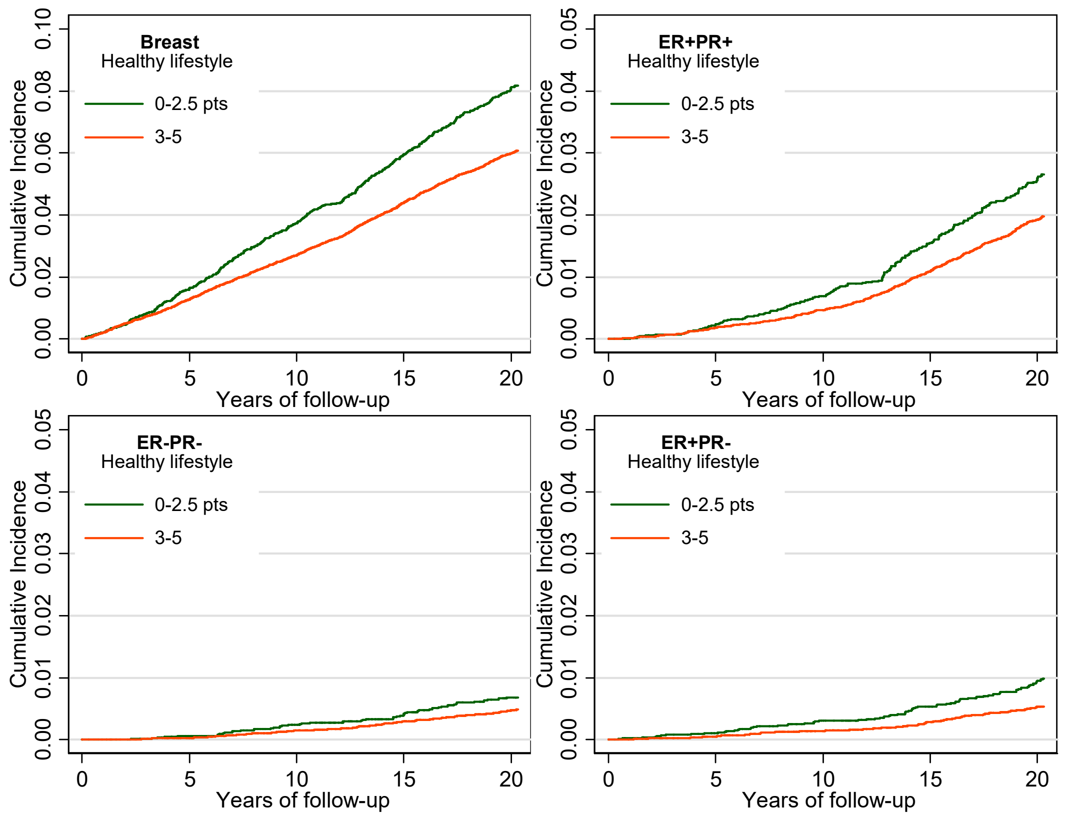

Supplementary Figure S4. Cumulative incidence of overall, ER+PR+, ER-PR- and ER+PR- breast cancer according to healthy lifestyle score (dichotomized), Netherlands Cohort Study.

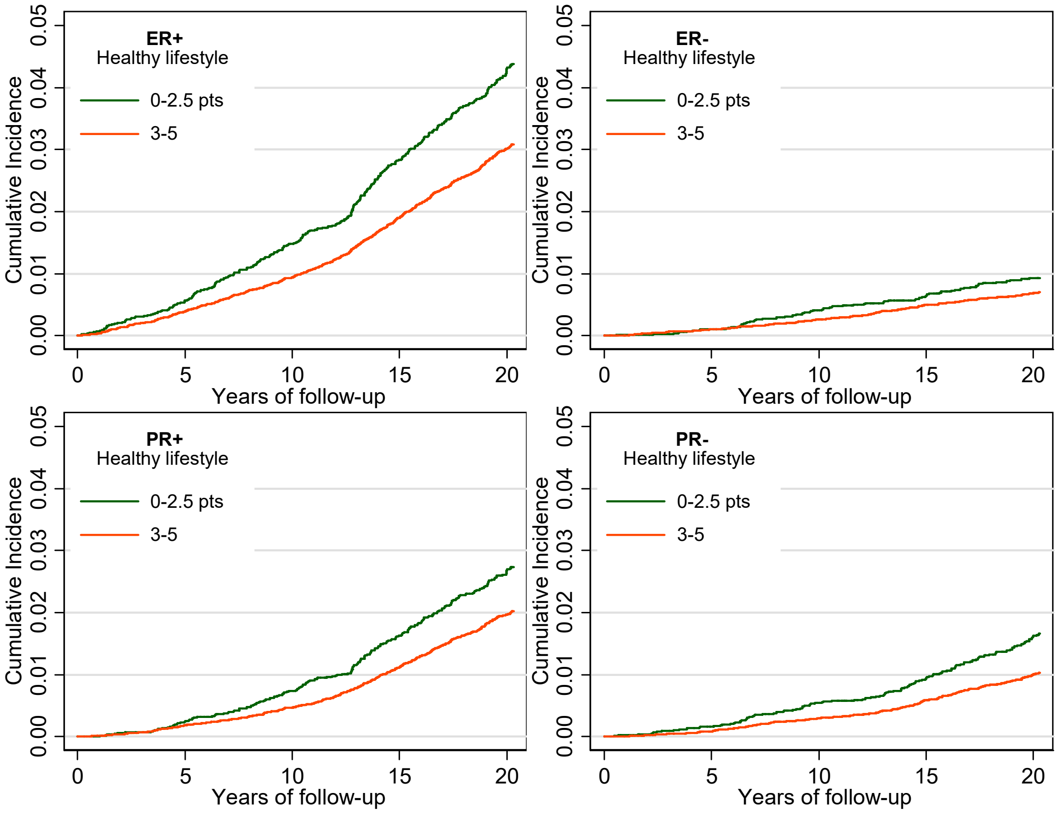

Supplementary Figure S5. Cumulative incidence of ER+, ER-, PR+ and PR- breast cancer according to healthy lifestyle score (dichotomized), Netherlands Cohort Study.

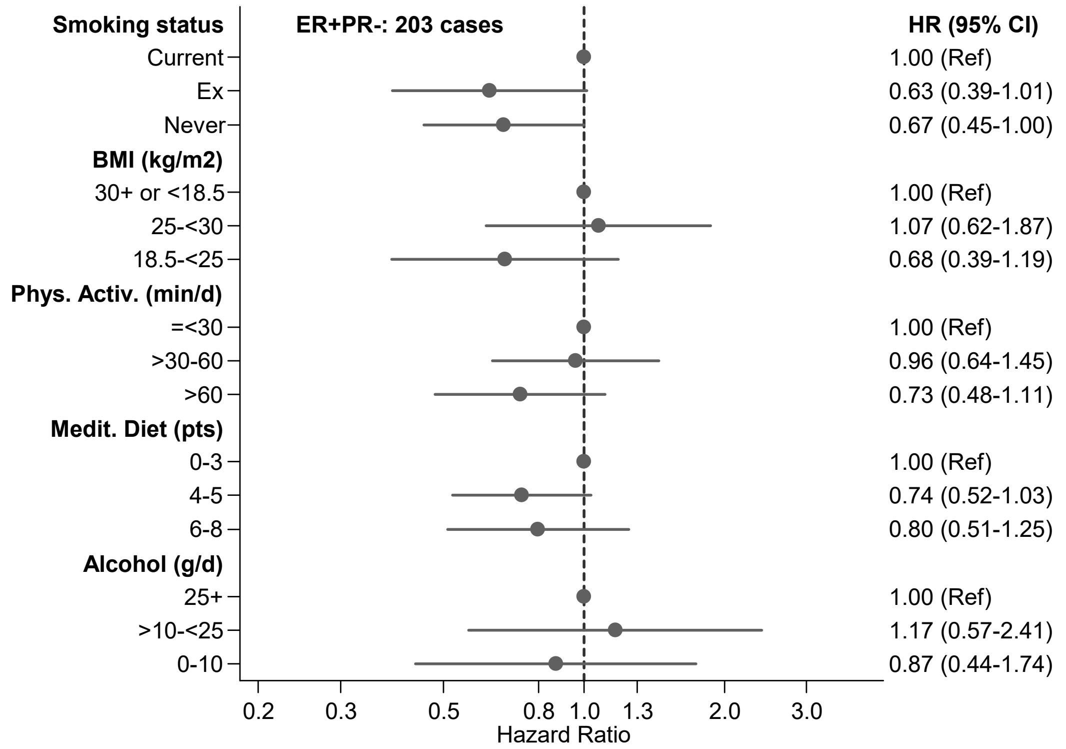

P-trend: Smoking status: 0.115; BMI: 0.020; Phys.Activ: 0.102; Medit.Diet: 0.183; Alcohol: 0.305

Supplementary Fig S6. Multivariable Hazard ratios and 95% CIs (error bars) for the association between risk of ER+PR- breast cancer and each of the component lifestyle factors of the HLS (with mutual adjustment for the other component lifestyle factors), Netherlands Cohort Study (NLCS).

Multivariable HRs were adjusted for: age at baseline (55-59, 60-64, 65-69 years), cigarette smoking frequency (number of cigarettes per day; continuous, centered) and duration (number of years; continuous, centered)), body height (continuous, cm), highest level of education (primary school or lower vocational, secondary or medium vocational, and higher vocational or university), family history of breast cancer in mother or sisters (no, yes), history of benign breast disease (no, yes), age at menarche (<12, 13-14, 15-16, >17 years), parity (nulliparous, 1-2, >3 children), age at first birth (<25, >25 years), age at menopause (<45, 45-49, 50-54, >55 years), oral contraceptive use (never, ever), postmenopausal hormone replacement therapy (never, ever), energy intake (continuous, kcal/day), other component lifestyle factors of the HLS.

Abbreviations: ER, Estrogen Receptor; PR, Progesterone Receptor; HLS, healthy lifestyle score.

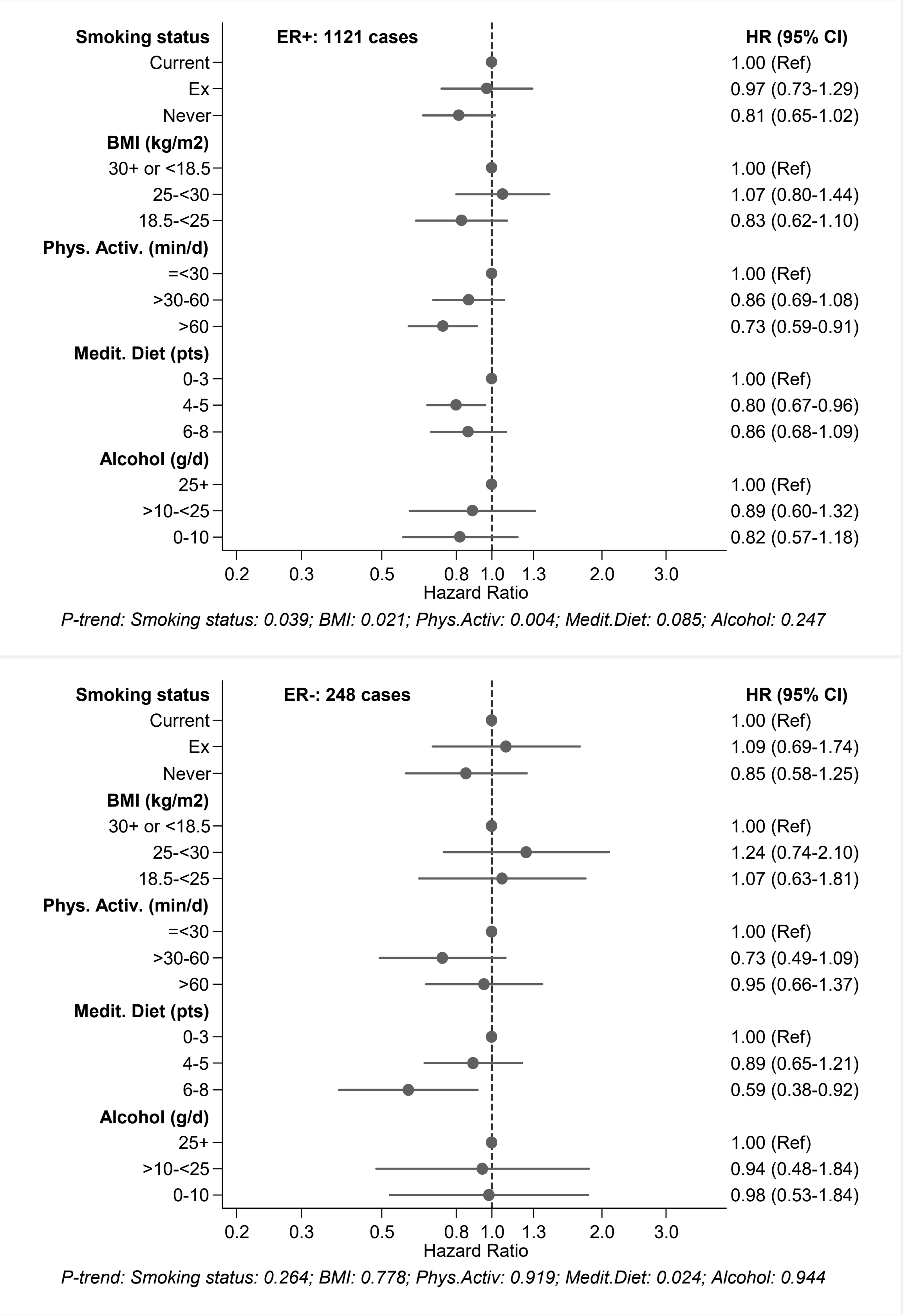

Supplementary Fig S7. Multivariable Hazard ratios and 95% CIs (error bars) for the association between risk of ER+ and ER- breast cancer, respectively, with each of the component lifestyle factors of the HLS (with mutual adjustment for the other component lifestyle factors, and adjustment for confounders as in Fig S3), Netherlands Cohort Study (NLCS).

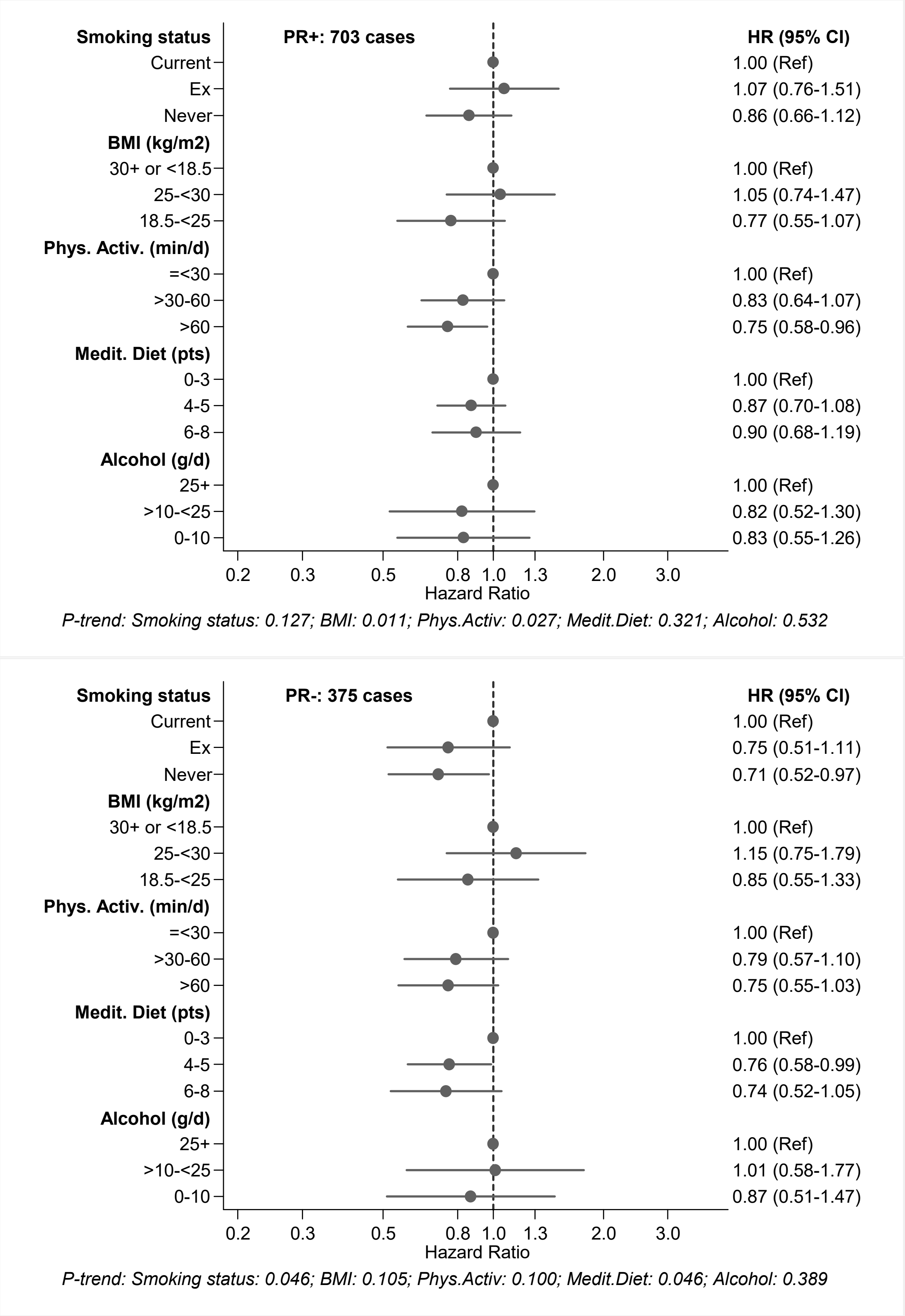

Supplementary Fig S8. Multivariable Hazard ratios and 95% CIs (error bars) for the association between risk of PR+ and PR- breast cancer, respectively, with each of the component lifestyle factors of the HLS (with mutual adjustment for the other component lifestyle factors, and adjustment for confounders as in Fig S3), Netherlands Cohort Study (NLCS).
